# Label-free assessment of pre-implantation embryo quality by the Fluorescence Lifetime Imaging Microscopy (FLIM)-phasor approach

**DOI:** 10.1038/s41598-019-48107-2

**Published:** 2019-09-13

**Authors:** Ning Ma, Nabora Reyes de Mochel, Paula Duyen Pham, Tae Yeon Yoo, Ken W. Y. Cho, Michelle A. Digman

**Affiliations:** 1 Department of Biomedical Engineering, University of California, Irvine, CA USA; 2 Laboratory of Fluorescence Dynamics (LFD), University of California, Irvine, CA USA; 3 Department of Developmental and Cell Biology, University of California, Irvine, CA USA; 4 Present Address: University of California San Francisco, School of Medicine, San Francisco, USA

**Keywords:** Embryology, Biomedical engineering, Optical techniques

## Abstract

Development of quantitative, safe and rapid techniques for assessing embryo quality provides significant advances in Assisted Reproductive Technologies (ART). Instead of assessing the embryo quality by the standard morphologic evaluation, we apply the phasor-FLIM (Fluorescence Lifetime Imaging Microscopy) method to capture endogenous fluorescent biomarkers of pre-implantation embryos as a non-morphological caliber for embryo quality. Here, we identify, under hypoxic and non-hypoxic conditions, the unique spectroscopic trajectories at different stages of mouse pre-implantation development, which is referred to as the developmental, or “D-trajectory”, that consists of fluorescence lifetime from different stages of mouse pre-implantation embryos. The D-trajectory correlates with intrinsic fluorescent species from a distinctive energy metabolism and oxidized lipids, as seen with Third Harmonic Generation (THG) that changes over time. In addition, we have defined a non-morphological Embryo Viability Index (EVI) to distinguish pre-implantation embryo quality using the Distance Analysis (DA), a machine learning algorithm to process the fluorescence lifetime distribution patterns. We show, under our experimental conditions, that the phasor-FLIM approach provides a much-needed non-invasive quantitative technology for identifying healthy embryos at the early compaction stage with 86% accuracy. The DA and phasor-FLIM method may provide the opportunity to improve implantation success rates for *in vitro* fertilization clinics.

## Introduction

Determining embryo quality during *in vitro* fertilization (IVF) is one of the most important steps toward successful pregnancy^1^. The standard non-invasive method to assess embryo quality and viability relies on the visual inspection of embryo morphology according to predefined criteria such as cell division patterns, the number of pronucleoli in cleavage stages^2,3^, and the physical characteristics of the blastocyst^4^. Assisted reproduction through morphological evaluation is labor intensive and highly dependent on the performance of individual physicians trained in these techniques. Development of more quantitative and objective means for assessing embryo quality that are simpler, safer, and faster could provide significant advantages in assisted reproduction by enabling single embryo transfers rather than the implantation of multiple embryos in order to increase the likelihood of a successful pregnancy.

**Figure 1:**
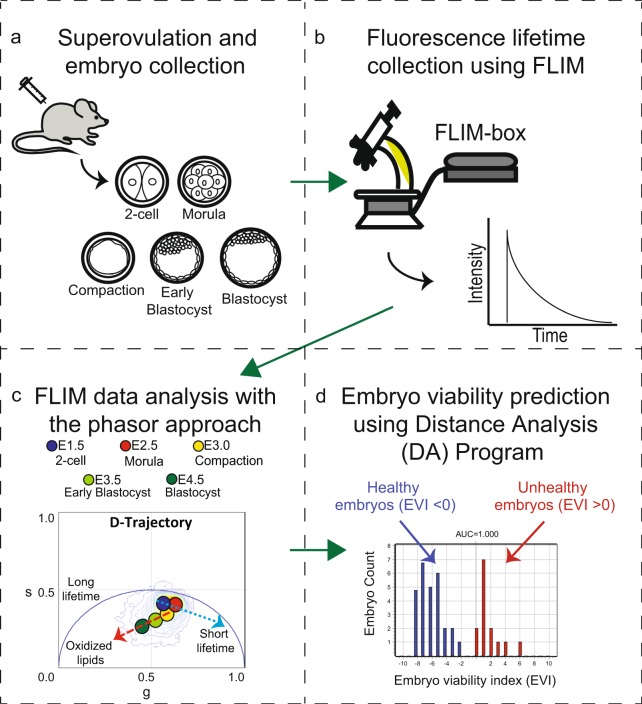
Schematic of the workflow of the experimental design. (**a**) We collected FLIM images of embryos from superovulated female mice at the following developmental stages: 2-cell, morula, compaction, early blastocyst, and blastocyst. **(b)** Intrinsic fluorescence lifetimes for each embryo are collected using a Zeiss 710 microscope coupled with a FLIM-box. **(c)** The FLIM data analysis of the pre-implantation mouse embryo development was performed using the phasor approach. **(d)** Distance Analysis (DA) program was applied to predict embryo viability.

Given the limitations of morphological evaluation, several technologies have been explored for the assessment of embryo viability. These include the measurement of metabolites in embryonic culture media, as well as genomic and proteomic profiling of the embryos themselves^5^. For example, spectroscopic approaches have been utilized to measure the number of metabolites such as pyruvate, lactate, and glucose in the media during embryo culture^6,7^. However, these approaches are time-consuming and require highly-trained personnel to analyze the complex data^8^. Both genomic and proteomic profiling are equally time consuming and can cause damage to the embryo during the procedure. Here, we apply the phasor-fluorescence lifetime imaging microscopy (FLIM) method and examine the dynamic endogenous biomarker (metabolites as described below) changes during preimplantation embryo development. Based on the quantifiable physiological property changes, we correlate the biomarker changes to the embryo viability (Fig. 1). This non-invasive phasor-FLIM analysis is sensitive, quick and intuitive.

FLIM produces an image, based on the exponential decay rates at each pixel from a fluorescent sample. The fluorescence lifetime of the fluorophore signal is measured to create the image via FLIM^9^ (Fig. S1A). When FLIM is coupled with two-photon excitation microscopy, molecules are excited at longer wavelengths (with lower energy photons). This prevents photodamage and allows deeper imaging, resulting in superior image quality^10^. Since endogenous molecules such as collagen, retinoids, flavins, folate and NADH (nicotinamide adenine dinucleotide) are fluorescent in live cells^11,12^, we can collect fluorescence lifetime data to identify these intrinsic fluorescent species. The contributions from these different biochemical species are indicators of an embryo’s biochemical property^13,14^. In our approach, we measure the fluorescent lifetime signal from integrated images acquired and transform the raw data using the Fourier transformation to the average arrival time of emitted photons in each pixel, represented by polar coordinates “g” and “s” in the transformation function^12^ (Figs 1c, S1A). This allows us to present the data in a two-dimensional graphical representation of the lifetime distributions, known as the phasor plot, for each pixel in the FLIM image (Fig. S1).

Here we have applied the phasor-FLIM approach to pre-implantation mouse embryos and have captured detailed data on their metabolic states at various developmental stages. At each stage, the mouse embryo displays a characteristic phasor-FLIM signature.

For the first time, we defined a unique graphical metabolic trajectory that correlates with energy metabolism and embryo development, which we call the developmental trajectory or “D-trajectory”. Initially, embryos uptake pyruvate as their main energy source^15^. As the embryos develop to later stages, the need for ATP increases in order to activate transcription for proliferation. Then, the embryos switch from glycolysis to oxidative phosphorylation, primarily using glucose as their energy source, which also changes the relative redox potential (NAD+: NADH ratio)^16^. The spectroscopic signatures from each of these changes are detected and can be used as criteria to identify healthy embryos at each stage in development. We find that the D-trajectory of pre-implantation embryos cultured in nutrient-deficient media deviates significantly from that of the normal media, indicating that lifetime trajectories can be used to detect metabolic alterations in embryos. We have identified a combination of mathematical parameters that are statistically different between healthy and unhealthy pre-implantation embryos based on machine learning information. Therefore, the phasor-FLIM approach provides an objective, non-invasive, and quantitative method to assess the quality of mammalian embryos.

## Results

### The lifetime D-trajectory of pre-implantation embryos

**Figure 2:**
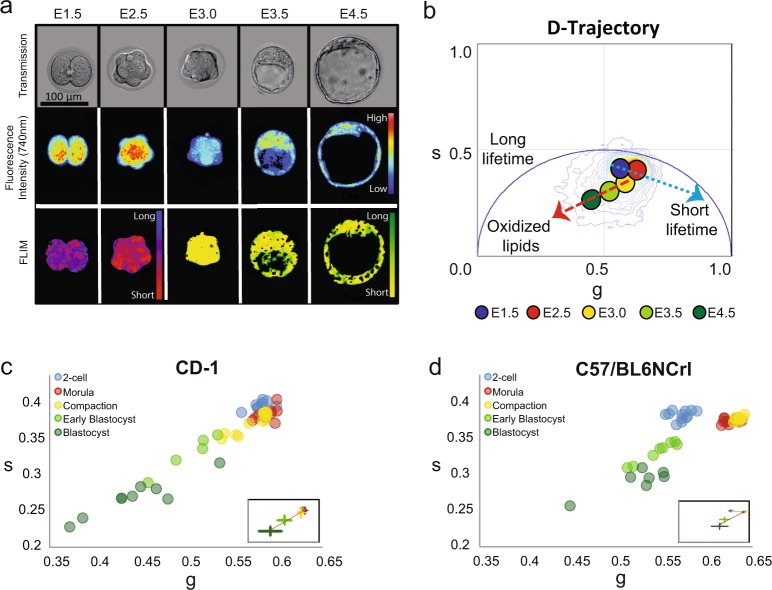
The lifetime trajectory of pre-implantation embryos correlates with embryonic development. (**a**) Transmission (top row), fluorescence intensity (middle row, 740 nm excitation) and FLIM (bottom row) images of representative pre-implantation CD1 mouse embryos at 2-cell (E1.5), morula (E2.5), compaction (E3.0), early blastocyst (E3.5), and blastocyst stage (E4.5). In the FLIM images, the pseudo color displays the fluorescence lifetime. **(b)** Phasor-plot of average fluorescence lifetime of CD1 embryos at the indicated developmental stages demonstrating the D-trajectory (D for development). A blue arrow indicates the fluorescence lifetime change from E1.5 to E2.5 and a red arrow shows the change from E3.0 to E4.5. **(c**,**d)** Scatter plots show the D-trajectory for CD1 and C57BL/6NCrl embryos. The small window shows the average and standard deviation of each stage. CD1: 2-cell (n = 29), morula (n = 11), compaction (n = 33), early blastocyst (n = 50) and blastocyst stage (n = 35); C57BL/6NCrl: 2-cell (n = 25), morula (n = 22), compaction (n = 21), early blastocyst (n = 38) and blastocyst stage (n = 42). (**c**) D-trajectory of CD1 embryos (2-cell, n = 8; morula, n = 8; compaction, n = 12; early blastocyst, n = 5; blastocyst, n = 8. and (**d**) D-trajectory of C57BL/6NCrl embryos (2-cell, n = 7; morula, n = 3; compaction, n = 17, early blastocyst, n = 8; blastocyst, n = 21). N = number of embryos analyzed.

Two different mouse strains (a non-inbred CD1 and an inbred C57BL/6NCrl) were used to acquire a comprehensive representation of the phasor-FLIM distribution patterns of embryos during pre-implantation development (Figs 2 and S2a). Fluorescent lifetimes of endogenous fluorescent species, excited at 740 nm, were collected at the 2-cell (E1.5), morula (E2.5), compaction (E3.0), early blastocyst (E3.5) and blastocyst stage (E4.5), and pseudo-colored according to the phasor coordinates (Fig. 2a,b). The phasor coordinates, which is the averaged fluorescent lifetime, of the 2-cell and morula stage embryos have a unique lifetime distribution pattern distinct from all other cell and tissue types measured (blue arrow, Fig. 2b)^11^. This unique phasor lifetime position may reflect special characteristics of totipotent cells, which mirror low oxygen consumption and preferential utilization of pyruvate oxidation^17^. On the other hand, compaction to blastocyst stages display average phasor coordinates typically observed in pluripotent cells (red arrow, Fig. 2b)^18,19^. We refer to this characteristic developmental time course lifetime distribution pattern as the developmental trajectory or “D-trajectory”. Phasor-FLIM lifetime distributions of individual embryos from both outbred and inbred mouse strains (Fig. 2c,d) follow the similar developmental trend D-trajectory. In order to examine whether genetic background of mice influences the D-trajectory, we compared the trajectories of both CD1 and C57BL/6NCrl strains (Fig. 2c,d). While the average lifetimes (g and s values) at specific embryonic stages are somewhat variable, the overall D-trajectory distribution (blue and red arrows) of C57BL/6NCrl is similar to that of CD1 mice. We conclude that the D-trajectory is a characteristic distribution behavior observed among pre-implantation mouse embryos. In addition, we have applied time-lapse FLIM imaging to individual embryos (n = 16), and continuously followed at 3-hour time intervals from 2-cell (E1.5) to blastocyst stage (E4.5) for approximately 60 hours. The *in vitro* developmental trajectory (Fig. S2b) of each embryo mirrors the D-trajectory (Fig. 2b, with blue and red arrows). Lastly, we have compared the phasor-FLIM developmental patterns between the pre-implantation embryos cultured under ambient (20.9% oxygen) and hypoxia condition (12.8% oxygen, trigas of 5% O_2_, 5% CO_2_, 90% N_2_ mixed with atmosphere) (Fig. S2c,d). After 4 hours of incubation, the 2-cell (E1.5), morula (E2.5), compaction (E3.0), early blastocyst (E3.5) and blastocyst stage (E4.5) embryos were subjected to FLIM collection of endogenous fluorescent species excited at 740 nm. The D-trajectories of embryos were similar between embryos grown under the ambient and hypoxic condition (Fig. S2d). Although we noticed slightly shifts towards the right for the hypoxic condition, presumably due to the higher glycolysis rate, the shifts for the s and g coordinates are not statistically significant. In sum, two combined lifetime trajectories (blue and red arrows) encompass the overall D-trajectory for normal pre-implantation embryo development.

Reactive oxygen species (ROS) plays a key role in cellular metabolism and homeostasis^20,21^ and ROS production has been linked to an increase in oxidized lipids^22^. The red arrow (the right to left-downward shift, Fig. 2b) in the D-trajectory is presumably due to an increasing fractional contribution of ROS as well as the oxidized lipids which have a fluorescence lifetime distribution of 7.89 ns and fall on the same published location (coordinates) of the semi-circle in the phasor plot (Fig. S1b)^12^. This behavior is consistent with the model that an increase in aerobic respiration and metabolism as well as β-oxidation during pre-implantation mouse development^12^ requires more efficient energy production from oxidative phosphorylation^23,24^. We have confirmed the presence of active ROS production with fluorogenic marker 2′, 7′-dichlorofluorescin diacetate (DCF-DA, also known as H_2_DCFDA) staining (Fig. S3).

**Figure 3:**
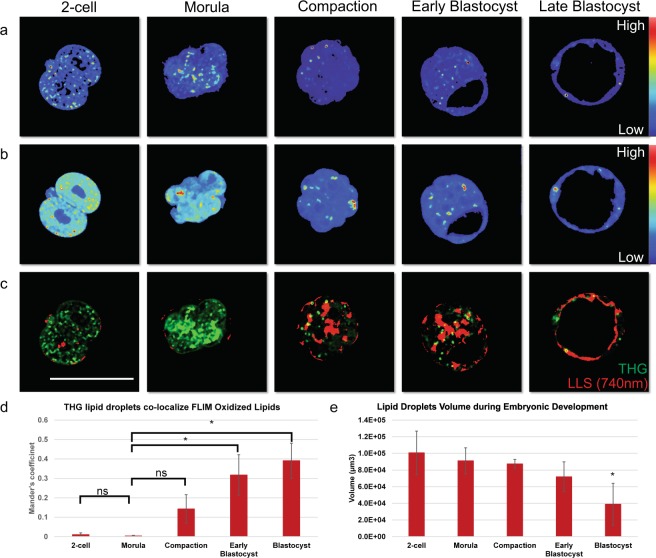
THG and intrinsic fluoresce signal show increasing oxidized lipids during embryonic development. (**a**) Representative third harmonic generation images and **(b)** FLIM images during pre-implantation embryonic development, 2-cell, morula, compaction, early blastocyst and blastocyst stage for the same field of view. From blue to red shows the intensity increase. **(d)** Representative optical sections show co-localization (yellow) of the lipid droplet signal (green) in THG images with long lifetime species (red) in FLIM images. Scale bar sets at 100 µm. **(d)** Mander’s coefficient of the co-localization results during embryo development which shows the proportion co-localization region of the THG channel and FLIM channel correspondence with long lifetime species-oxidized lipids. 2-cell (n = 5), morula (n = 3), compaction (n = 3), early blastocyst (n = 4) and blastocyst stage (n = 3). Student t-test results (p-value) for morula to 2-cell, compaction, early blastocyst and blastocyst are 0.1923, 0.0823, 0.0091, and 0.0174 respectively. (**e**) Lipid droplets volume characterization during pre-implantation embryo development. 2-cell (n = 5), morula (n = 5), compaction (n = 4), early blastocyst (n = 4) and blastocyst stage (n = 6). Student t-test results (p-value) for morula to 2-cell, compaction, early blastocyst and blastocyst are 0.5066, 0.6367, 0.1416, and 0.0072 respectively.

In order to better characterize the lipid droplets distribution during embryonic development, we have employed third-harmonic generation (THG) microscopy imaging (Fig. 3) with Deep Imaging Via Emission Recovery (DIVER) microscope (Fig. 3). The interfaces heterogeneity can be detected with the third order nonlinearity χ^3^. Given that the process is ultra-fast for structures with THG signals, the lifetime is approximately zero. Figure 3a shows the representative THG intensity images acquired in the same field of view as that of the FLIM images of Fig. 3b. The phasor plot of the THG images appears at the coordinate of s = 0 and g = 1. Movie S1 shows the 3D structure of the lipid droplets of embryos from different stages^25^. Furthermore, we quantify the co-localization correlation of the long lifetime specie in the FLIM images (red) with the lipid droplets (green) in THG images (Fig. 3c,d). During embryonic development, the oxidized lipid signature, color-coded in red for the long lifetime species, (same direction as red arrow in Fig. 2b) accumulated. The Mander’s split co-localization correlation coefficients increase from 0.0099 to 0.3907 (where a coefficient of 1 is perfect correlation and 0 is complete lack of correlation) with embryonic development, suggesting that the phasor-FLIM distribution changes during these stages are due to increased lipid accumulation. We also characterized the lipid droplets distribution during embryonic development using the 3D THG image (Fig. 3a,e). Cleavage stage embryos have a large amount of small, densely packed lipid droplets, whereas post-cleavage stage embryos have large lipid droplets of the low density. The dramatic changes for both the lipid oxidation and lipid volume size start after compaction stages. These findings demonstrate that the dynamic difference in lipid oxidation can be detected by phasor-FLIM.

### Fluorescence lifetime trajectories reveal metabolic states of pre-implantation mouse embryos

The D-trajectory is complex because it is composed of lifetimes from various endogenous fluorescent biochemical species. We first hypothesized that the major component responsible for the shifts in the D-trajectory is intracellular NADH changes based on its fundamental role in energy production during embryogenesis. To test this hypothesis, we first measured the metabolic activity of intracellular NADH^26^. The bound form of NADH is linked to energy production through oxidative phosphorylation, whereas the free form of NADH is associated with glycolysis^27^. The phasor coordinates of free NADH maps on the right side of the plot with a lifetime of 0.38 ns and the protein bound form of NADH (bound with lactate dehydrogenase) maps on the left at 3.4 ns (Fig. S1b). This lifetime distribution of the free and bound forms of NADH in the phasor plot was previously described as the metabolic or M-trajectory^11^.

**Figure 4:**
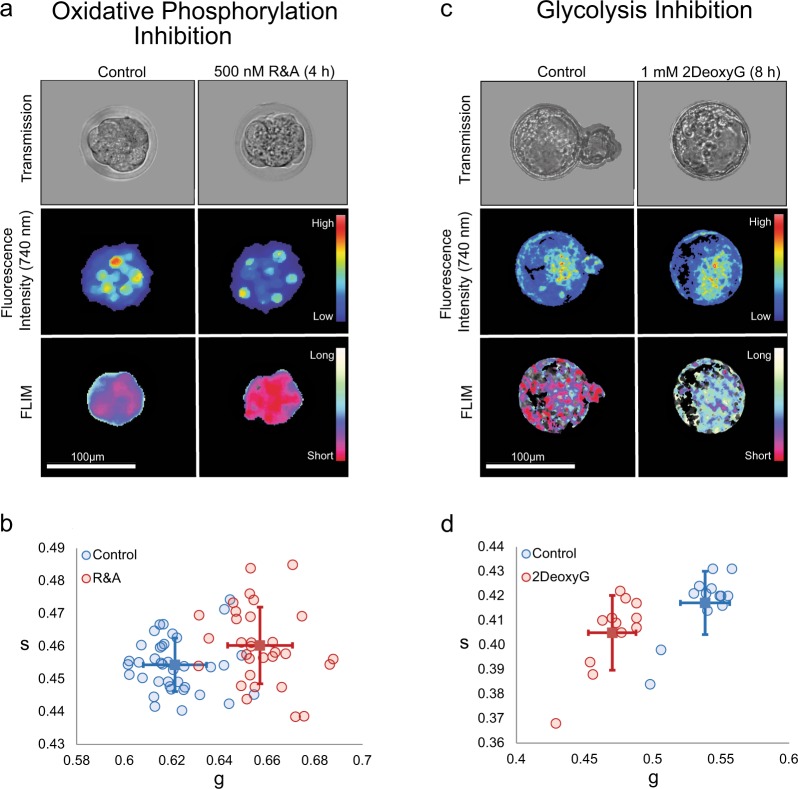
Fluorescence lifetime trajectories reveal metabolic states of pre-implantation mouse embryos. (**a)** Transmission (top), fluorescence intensity (middle) and FLIM (bottom) images for control and 4-hour rotenone and antimycin A (R&A) treated embryos. Note a shift from long to short lifetimes (blue to red in FLIM image). **(b)** g and s values of control and R&A-treated embryos for individual embryos. Blue circles are controls (n = 38), red circles are R&A-treated embryos (n = 31), and solid squares and the error bars in the figures means the average and variation of each group (student t-test for g value: p-value = 2.86E-16). FLIM images indicate a rightward shift from long to short lifetimes. **(c)** Transmission (top), fluorescence intensity (middle) and FLIM (bottom) images for control and 2DeoxyG-treated embryos. Note a shift from long to short lifetimes (red to white in FLIM image). **(d)** g and s values of control and 2DeoxyG-treated embryos. Blue squares are controls (n = 12), red circles are 2DeoxyG -treated embryos (n = 13), and the average of each group can be found in the solid colored squares (student t-test for g value: p-value = 3.88E-09). Fluorescence and FLIM images indicate a leftward shift from long to short lifetimes.

Next, embryos were treated with known biochemical inhibitors of oxidative phosphorylation and glycolysis^27^. Oxidative phosphorylation was inhibited at the early compaction stage with a cocktail of rotenone and antimycin A (R&A) (500 nM) by inhibiting complex I and complex III of the electron transport chain. Embryos were imaged after a 4-hour culture period (Fig. 4a). The FLIM images showed increased fractional contributions of free NADH (shorter lifetimes) when compared to controls (Fig. 4a). This shift towards glycolytic metabolism is seen in a dose-dependent manner (Fig. S4), indicating that embryos cultured in R&A have decreased oxidative phosphorylation activities (Fig. 4a,b). We also cultured the early blastocyst stage embryos in 1 mM 2-Deoxy-D-Glucose (2DeoxyG), an analog of glucose, to inhibit glycolysis (Fig. 4c). The glucose analog treatment shifted the phasor-FLIM distribution to longer lifetime (an increase of bound NADH) (Fig. 4c), which correlates with a decrease in glycolysis (Fig. 4c,d). These findings suggest that the source of the changes seen in the phasor coordinates throughout the pre-implantation stages in the D-trajectory is in part due to the contribution from metabolic shifts of NADH.

### FLIM does not disrupt embryonic development under 10 mW laser power

In order to ensure the safety of the FLIM imaged embryos, we determined the optimum laser power to avoid DNA damages^28^, while allowing the rapid and robust acquisition of the FLIM signal on mouse pre-implantation embryos. We exposed 2-cell (E1.5) and morula (E2.5) stage CD1 and C57/BL6NCrl embryos to varying laser powers (1.5, 3.5, 10, and 15 mW) and examined the effect on the developmental progression of embryos until the blastocyst stage (Figs S5, 6). In order to capture FLIM-signals of embryos taken with 1.5 mW laser power, 4 times longer exposure time was required than the embryos collected at 3.5 mW, 10 mW, 15 mW laser powers due to their low signal to noise ratio. The majority of embryos exposed to 1.5 mW and 3.5 mW laser power developed to the blastocyst and there were no significant differences between the control (non-imaged) and embryos imaged at the 2–4 cell stage or morula-compaction stage, irrespective of strain differences (CD1 or C57BL/6NCrl) (Figs S5, 6). However, at 10 mW, approximately 20% and 35% of CD1 embryos imaged at the 2-cell and compaction stages, respectively, fail to progress to the blastocysts. At 15 mW, nearly 50% of CD1 and C57BL/6NCrl embryos imaged at the 2-cell stage were arrested before the compaction stage, while approximately 30% of CD1 and 12% of C57/BL6NCrl embryos imaged at the compaction stage failed to develop to blastocysts. We conclude that CD1 embryos are more sensitive to the laser damage, and 3.5 mW is the ideal laser power for our FLIM analysis.

Next, we examined the activation of the DNA repair pathway in the embryo by conducting immunofluorescence staining for anti-phosphorylated Histone 2AX (H2AXs139), a novel marker for DNA-double strand breaks^29,30^. Both the non-imaged control and FLIM-imaged embryos were indistinguishable and did not show any signs of DNA repair pathway activation at 3.5 mW (Fig. S7a). However, embryos exposed to 1.5 mW laser power, which required longer laser exposure time (12 minutes, instead of ~3 minutes) showed the sign of DNA damage (Fig. S7a).

Next, we examined the effects of FLIM on the rate of pregnancy. Specifically, FLIM-imaged and control BRE-gal embryos (a reporter mouse line responding to the endogenous levels of BMP signaling during development)^31^ at E2.5 were allowed to develop to the early blastocyst stage (E3.5) and control and FLIM-imaged embryos were implanted into females. E18.5 embryos were collected through caesarean section (C-section) and counted for the implantation efficiency (Fig. S7b, and Supplementary Table 1). The average live birth rates were 49% for FLIM-imaged group and 43% for the non-imaged BRE-gal control group based on three independent experiments (Supplementary Table 1). Student t-test reveals that there is no statistically significant difference between FLIM-imaged and non-imaged group. We conclude that FLIM imaging of the morula stage embryo at 3.5 mW excitation is safe to use and employed in the subsequent experiments.

### FLIM distinguishes pre-implantation embryos under stress conditions

Given that early cleave stage embryos utilize aspartate, pyruvate, and lactate for energy metabolism^32^ we tested whether the unique lifetime distribution patterns of an embryo cultured under altered physiological states can be detected by the changes in spectroscopic distributions of phasor-FLIM.

**Figure 5:**
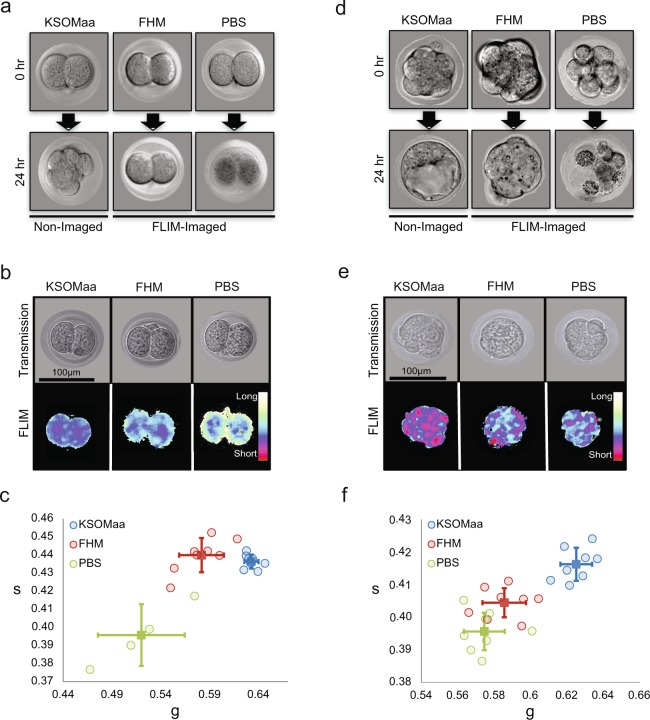
Deviation of intrinsic lifetime trajectory of embryos cultured in nutrient-depleted media. **(a)** Transmission images of embryos collected at the 2-cell stage and cultured in KSOMaa, FHM, or PBS for 24 hours. **(b)** Representative transmission and FLIM images of embryos in KSOMaa, FHM, or PBS for 4 hours. **(c)** Scatter plot of g and s lifetimes collected from a group of embryos cultured in KSOMaa (n = 10), FHM (n = 10) and PBS (n = 4) for 4 hours. p-value = 0.0002** and 0.01* (student t-test of g value) for the FHM and PBS group compare with KSOMaa group. **(d)** Transmission images of embryos collected at the compaction stage and cultured in KSOMaa, FHM, or PBS for 24 hours. **(e)** Representative transmission and FLIM images of embryos in KSOMaa, FHM, or PBS for 4 hours. **(f)** Scatter plot of g and s of lifetimes collected from a group of embryos cultured in KSOMaa (n = 8), FHM (n = 8), and PBS (n = 8). p-value = 9.29E-06** and 3.21E-07** (student t-test of g value) for the FHM and PBS group compare with KSOMaa group.

We cultured 2-cell and morula stage embryos in standard mouse embryo culture media (KSOMaa), flushing and holding media (FHM: DMEM-pyruvate free with HEPES), and saline solution (PBS). Brightfield images and FLIM data were collected at 4 hours and 24 hours after the treatment (Fig. 5). The FLIM data were collected once at the first time-point (4 hours). Two-cell stage embryos cultured under KSOMaa, FHM and PBS were morphologically normal (Fig. 5a, top). However, embryos in high-stress conditions (FHM and PBS) show distinct lifetime distribution patterns on the phasor-plot when compared to those of KSOMaa cultured embryos (Figs 5b,c, S8a). Subsequently, we find that the embryos under high-stress conditions fail to cleave normally and remain at the 2-cell stage, unlike KSOMaa controls (Figs 5b, S8a). We performed the similar analysis using compaction stage embryos and found that within a few hours under high-stress culture conditions, the phasor-FLIM lifetime trajectories of embryos deviate from those cultured in KSOMaa even before the embryos show any signs of abnormal morphology (Figs 5d–f, S8b). The cell division in FHM and PBS cultured embryos also slowed down significantly (Fig. S8b). We conclude that phasor-FLIM is a sensitive method to detect the changes in embryo metabolism upon cellular stress.

### Derivation of the embryo viability index (EVI) for assessing the developmental potential of the pre-implantation embryo

**Figure 6:**
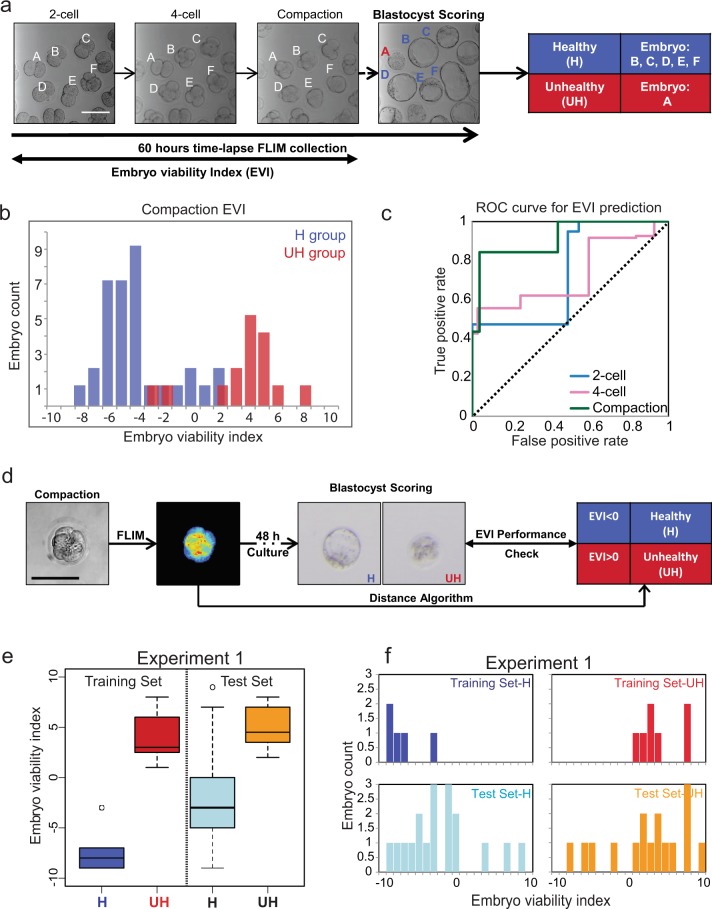
Derivation of the embryo viability index (EVI) gauging embryo quality. **(a)** Schematic of our experimental setup. Individual embryos (A–F) were followed from the 2-cell to blastocyst stage and classified as healthy (H) and unhealthy (UH) group according to their morphology at E4.5. (**b**) Histogram of embryo viability index (EVI) of early compaction embryos from one representative experiment (H group, n = 37; UH group, n = 27). The blue and red bars represent the embryo condition determined as healthy and unhealthy at ~60 hours after FLIM imaging at the pre-compaction stage. (**c**) Receiver operating characteristic (ROC) curve shows the performance of the binary classification model developed from lifetime distribution patterns of early developmental stage embryos (2-cell, 4-cell, and early compaction stage). The area under a curve for each stage is 0.739 (2-cell), 0.728 (4-cell) and 0.916 (early compaction). The dashed line in the diagonal is presented as a random bi-classification model. **(d)** Schematic of FLIM-Distance Analysis Pipeline. **(e)** Box-whisker plots of experiment 1 showing a training set of healthy (n = 5) and unhealthy (n = 7) groups and tested unknowns of healthy (n = 18) and unhealthy (n = 16) embryos. **(f)** Bar graph of embryo viability index of experiment 1. Training set H is in navy, training set UH is in red. Testing set H is in light blue, and Testing set U is in orange.

The phasor distribution analysis of pre-implantation mouse embryos allows us to distinguish between normal and highly stressed embryos (Fig. 5). Therefore, we determined whether the developmental potential of pre-implantation embryos is predictable through phasor-FLIM analysis. We first performed time-lapse phasor-FLIM imaging of embryos from the 2-cell stage for ~60 hours to identify the most desirable stage to predict the developmental potential of embryos (Fig. 6a, Movie S2). At the end of the 60-hour culture period, we classified embryos as healthy (H) if they reached the normal full expanded blastocyst stage showing a tightly packed ICM and cohesive epithelium shaped TE cells, or not healthy (NH) if embryos were arrested before reaching the blastocyst stage or displaying abnormal morphological features such as having a smaller blastocoel cavity and/or irregular cell boundary at the blastocyst (Fig. 6a). We then applied the distance analysis (DA) algorithm^33^ to identify key spectroscopic parameters that could differentiate healthy (H) from unhealthy (UH) embryos by machine learning. Using the DA algorithm, the 3D phasor histogram was separated into 4 sections based on the phasor coordinates (g, s) intensity, from which, 6 parameters were extracted from each section, generating a total of 24 parameters (see Methods). The healthy embryos (H group) were used as the control set and the unhealthy embryos (UH group) were used as the sample set. Each of these sets included images from multiple embryos from each stage in development. Next, we calculated the average and variance of the training set, which includes two groups (H and UH), and weighted 20 parameters (g, s, the secondary moment a, b and angle from 4 sub-layers, intensity excluded) in each set from 3D phasor plot. After optimizing the weights to maximize the difference between unhealthy and healthy group embryos, we applied these weights to index a new score called the EVI or Embryo Viability Index (Methods). This partition metric defines the degree of separation of the test embryos from the average of the training set where −1 to −10 are unhealthy embryos, and +1 to +10 are healthy embryos.

Next, we examined the DA data from 2-cell, 4-cell, and the early compaction stage to determine the best binary classification model using receiver operating characteristic (ROC) curves (Figs 6b,c, S9a,b). We have classified the embryos predicted to be healthy in positive values (EVI < 0, in blue), and embryos predicted to be unhealthy in negative values (EVI > 0, in red). The plot of true positive rates against false positive rates gives an area under the ROC curve (AUC) for 2-cell, 4-cell, and the early compaction stage embryos, which were 0.739, 0.728, and 0.916, respectively. We conclude that the spectroscopic characteristics of the early compaction stage embryos (prediction accuracy with the highest AUC) possess the best parameters for separating embryos that will develop into normal blastocysts (Figs 6b,c, S9a,b).

An embryo viability prediction pipeline was developed based on the DA of phasor-FLIM images of the early compaction stage embryos (Fig. 6d). We have FLIM imaged embryos at the early compaction stage and all of these embryos were allowed to develop to the blastocyst equivalent stage. The resulting embryos were classified as H or UH. We then selected a small number of healthy (H) and unhealthy (UH) embryos and obtained an EVI training data set. The remaining unselected embryos were also subjected to the DA program as “unknowns” (test set) to test the predictability of EVI. In experiment 1, we followed the development of 35 morphologically healthy-looking early compaction stage embryos (pooled from 4 mating pairs), until the blastocyst stage (Fig. 6e,f). Of the 34 embryos, 18 developed to normal blastocysts and thus assigned as healthy (H), and 16 embryos that failed to reach the blastocyst were assigned as unhealthy (UH). When we applied EVIs that were determined by the training set, 83.3% of healthy embryos (15 out of 18 embryos) and 75.0% of unhealthy embryos (12 out of 16 embryos) were correctly predicted by EVI (Fig. 6e,f). Subsequently, we performed another 4 biologically independent experiments using a total of 134 embryos and the results are shown in Supplementary Table 2 and Supplementary Figure 6c, d. We achieved 85.9% accuracy (n = 134) where a total of 88.5% healthy embryos (n = 96) and 73.7% unhealthy embryos (n = 38) were identified. Based on the results, we conclude that the DA program is able to predict the development potential of pre-implantation embryos at the early compaction stage.

## Discussion

Here we report that the phasor-FLIM represents a promising new approach for assessing the quality of pre-implantation mouse embryos. First, we have applied the phasor-FLIM analysis to capture developmental states during pre-implantation development. The spectroscopic trajectory, which we are calling the “D-trajectory” (D for development), is attributed to a combination of metabolic fluorescent species and production of ROS in conjunction with oxidized lipid metabolism within the embryo (Fig. 2c,d), and this trajectory correlates well with other measurements of embryonic development. Second, the intrinsic lifetime trajectory of pre-implantation embryos cultured in nutrient-deficient media deviates from the normal lifetime distribution, indicating that the lifetime trajectory can be used to detect metabolic changes in embryos. Third, we have applied the DA program that uses spectroscopic parameters from 3D phasor histograms of embryos and shown that EVI is a non-morphological, quantitative index that can provide useful information on the quality of pre-implantation embryos.

Other spectroscopic technologies have emerged as a non-invasive means of revealing embryo viability via detection of various metabolic states of common molecules associated with embryo development. Raman, near-infrared, Nuclear Magnetic Resonance (NMR), and Fourier-transform infrared spectroscopy can also detect the metabolic states of pyruvate, lactate, glucose, and oxygen during pre-implantation mammalian development^34–36^. However, at present time these technologies suffer from a number of shortcomings. It is challenging for these approaches to analyze the data in the short time window needed for the host transfer of embryos. The data analyses are technically demanding and may not be intuitively obvious for the general clinical use. The technologies require fluid samples collected from the embryo culture media and the data are inherently noisier. Nonetheless, in the future, with improvements, these spectroscopic approaches are likely to provide physicochemical parameters that will be useful in quantitating the influence of ovulation induction, oocyte retrieval, and *in vitro* culture procedures.

Development of qualitative and objective means for assessing embryo quality and viability that are safer and faster will provide significant advances in IVF and animal breeding facilities. If phasor-FLIM is to be applied for diagnostic purposes, it will be crucial to establish that the procedure does not perturb gene expression after the procedure. To date, embryos subjected to phasor-FLIM analysis appeared to be morphologically normal, and we did not detect signs of apoptosis or aberrations in nuclear morphology. However, it is possible that the phasor-FLIM procedure causes other alterations that cannot be easily detected by these morphological criteria. In the future, it will be important to perform additional molecular characterizations (i.e., DNA sequencing) to eliminate the possibility. In addition, our future experiments will include the assessment of implantation efficacy of these indexed embryos. Overall, this work has the potential to improve our understanding of energy metabolism in developing mammalian embryos and advance the ART field directly.

## Methods

### Animals

Animals were treated according to standards set by UC Irvine’s University Laboratory Animal Resources (ULAR). CD1 and C57BL/6NCrl females were purchased from Charles River Laboratories. All animal procedures were performed with strict adherence to National Institutes of Health office of laboratory animal welfare (NIH OLAW) and Institutional Animal Care and Use Committee (IACUC) guidelines.

### Ethics statement

Mice used for these experiments were used in accordance with the regulations overseen by the University of California Irvine Institutional Animal Care and Use Committee (IACUC) who assures that the use of live, vertebrate animals in research, testing, teaching or related activities is scientifically justified in accordance to Federal regulations and accreditation standards. All the techniques and procedures in this project have been refined to provide for maximum comfort and minimal stress to the animals. We confirm all experiments were performed in accordance to the guidelines and regulations by the protocol animal welfare assurance number approved by University of California Irvine Institutional Animal Care and Use Committee: A3416-01.UCI has been accredited by the Association for the Assessment and Accreditation of Laboratory Animal Care, International (AAALAC) since 1971.

### Pre-implantation mouse embryo collection

Females at 21-24 days old were superovulated with pregnant mare serum gonadotropin (PMSG, Sigma) and 48 hours later with human chorionic gonadotropin (hCG, Sigma). Matings were set each evening after hCG injections. The following morning a vaginal plug was considered 0.5 days post fertilization and embryos were collected at desired stages by flushing oviducts or uterine horns. For our time course collection (intrinsic fluorescence FLIM and THG measurements) superovulation and matings were staggered and all the embryos were collected the same day except late blastocysts (E4.5) were generated by dissecting at E3.5 (one day before imaging) and cultured till next day.

### Embryo culture

Embryos were cultured at 12.8% for hypoxia condition or 20.9% O2 (measured using Neofox oxygen sensor), with 5% CO2 in nitrogen balance at 37 °C. The drop size used was on average ~10 embryos/20 µl drop (1 drop per dish) except for the prediction test, where the drop size was on average 1 embryo/3 µl drop (~10 drops/dish). Embryos were cultured and imaged on Matek Glass bottom Dishes (P35G-1.5-14-C). Single embryo cultures were used for embryo viability prediction (of 1 embryo per 3 µl of KSOMaa) to prevent the mobility of embryos and provide stable environment, to avoid ROS accumulation or influence of neighboring embryos, and to create a library for prediction and embryonic developmental potential. All other experiments were performed on group cultures ~10 embryos/20 µl drop (1 drop per dish).

### Fluorescence lifetime imaging microscopy (FLIM)

Fluorescence lifetime images of the pre-implantation embryos were acquired on Zeiss LSM710 (Carl Zeiss, Jena, Germany), a multi-photon microscope coupled with a Ti: Sapphire laser (Spectra-Physics Mai Tai, Mountain View, CA) with 80 MHz repetition rate. The FLIM data detection was performed by the photomultiplier tube (H7422p-40, Hamamatsu, Japan) and a320 FastFLIM FLIMbox (ISS, Champaign, IL). The pre-implantation mouse embryos were excited at 740 nm; an average power of ~3.5 mW was used as previously in live cells and tissue^37^. A Zeiss EC Plan-Neofluar 20x/0.5 NA objective (Cart Zeiss, Jena, Germany) was used. The following settings were used for the FLIM data collection: image size of 256 × 256 pixels, scan speed of 25.21 µs/pixel. A dichroic filter at 690 nm was used to separate the fluorescence signal from the laser light. And the emission signal is split with 496 nm LP filter and detected in two channels using a band pass filter 460/80 and a 540/50 filter. Every FLIM image was acquired for 50 frames of the same field of view with 256 × 256 per frame. Only the blue channel (460/80) data was used for this study. FLIM calibration of the system was performed by measuring the known lifetime of a fluorophore coumarin 6 (dissolved in ethanol), which has a known fluorescence lifetime of τ = 2.5 ns^38,39^. Embryos were kept in standard culture conditions, 37 °C and at 5% CO_2._ FLIM data were acquired and processed by the SimFCS software developed at the Laboratory of Fluorescence Dynamics (LFD).

### Converting FLIM data onto phasor coordinates

All FLIM images are transformed onto the phasor plot by the equations below. The g and s coordinates are generated from the fluorescence intensity decay of each pixel in the FLIM image using the following Fourier transformation equations (Fig. S1A).

$$\begin{array}{c}{g}_{i}(\omega )={\int }_{0}^{\infty }I(t)\cos (\omega t)dt/{\int }_{0}^{\infty }I(t)dt\\ {g}_{i}(\omega )={\int }_{0}^{\infty }I(t)\sin (\omega t)dt/{\int }_{0}^{\infty }I(t)dt\end{array}$$

Thus, the phasor approach is a fit-free analysis of FLIM imaging, and the g and s coordinates represent the decay curve at each pixel of the image. Therefore, a phasor analysis transforms complicated spectrum and decay of every single pixel into a unique position on the phasor plot.

### Third harmonic imaging

The third harmonic generation images and the associated FLIM images of the same field of view were collected using the homebuilt Deep Imaging via Enhanced-Photon Recovery (DIVER) microscope. DIVER microscope is an upright laser scanning microscope, the unique feature is the application of wide photocathode area detector which allows collection of photons from a wide area and angle for high efficiency. The third harmonic generation images and intrinsic fluorescence FLIM images were collected using 40x water immersion objective (Olympus Plan Apo) with 1040 nm and 740 nm excitation respectively. And UG11 and Blue5543 filters were used for THG and endogenous fluorescence FLIM images collection. An a320 FastFLIM FLIMbox (ISS, Champaign, IL) was used to transfer the data to the phasor plot. Rho110 was used for calibration with known lifetime τ = 4 ns^39^.

### Inhibition of oxidative phosphorylation and glycolysis

Embryos were placed in 25 µl microdroplets of KSOMaa (Invitrogen) with the appropriate inhibitors covered in mineral oil (Sigma). Both of the two chemical inhibitors, rotenone and antimycin A cocktail (R&A) and 2-Deoxyglucose (2DeoxyG) were dissolved in KSOMaa. For R&A, we prepare the inhibitor to perform dose dependence measurements for a final concentration of 100 nM and 500 nM. For 2DeoxyG the inhibitor has a final concentration of 1 mM. KSOMaa was used as a solvent and culture media for the control group and treatment group embryos.

### H2AXs139 staining

CD1 and C57BL/6NCrl post-imaged embryos are rinsed with Tyrode’s acid (Sigma) 3 times and placed in holding and flushing media for 5 minutes to allow embryos to acclimate before 30-minute fixation in 4% paraformaldehyde on ice. Embryos were permeabilized using 0.2% Triton X-100 (Fisher). And then embryos were incubated with H2AXs139 (Genetex) at 1:1000 for 1 hour at room temperature. Embryos were rinsed in 1X PBT three times and then stained with AlexaFluor555 at 1:200. Embryos were rinsed in 1X PBS three times before processing for the Hoechst (Sigma) staining for 10 minutes to stain the DNA. Finally, embryos were rinsed and imaged in 1X PBS using 780 Zeiss microscope and Zen 2012 software.

### Embryo implantation and C-section at E18.5

CD-1 female mice were mated with vasectomized males to generate pseudo pregnant females timed to E3.5 for implantation. E2.5 embryos were collected and imaged, and implanted at E3.5. In each experiment, embryos were randomized before imaging to non-imaged and FLIM-imaged groups. After imaging, non-imaged embryos and FLIM-imaged embryos were randomized. The technician transferring the embryos was blinded to which embryos were imaged or non-imaged. Twelve to sixteen embryos were implanted into the left and right uterine horn of pseudo pregnant females. E18.5 embryos were collected through C-section and counted for the implantation efficiency. Genotyping was done for experiments that used BRE-gal^+/−^ embryos to differentiate between WT embryos to BRE-gal^+/1^ embryos. Embryos were genotyped with Tissue Direct Phire PCR Kit with the following primers: (LacZ band) Fwd: 5′ ATG AGC GTG GTG GTT ATG C 3′ Rev: 5′ GAT GGT TCG GAT AAT GCG 3′ (Hprt band) Fwd: 5′ AAG CCT AAG ATG AGC GCA AG 3′ Rev: 5′ AAG CGA CAA TCT ACC AGA GG 3′.

### DCF-DA Staining

Embryos are rinsed in Acid Tyrode 3×, washed in KSOMaa 3×, transferred to 5 uM DCF-DA in 1X PBS. Embryos were incubated in DCF-DA solution for 25 min at 5% CO_2_ and 37 °C. Embryos were then transferred to Hoechst stain solution for 8 min. Then embryos were placed in KSOMaa and imaged with LSM780 at 5% CO_2_ at 37 °C.

### Antibody Staining Image analysis for cell number calculation

We used a 3D segmentation pipeline (as previously described)^40^ to do a 3D reconstruction of embryos and conduct cell number analysis.

### Time-lapse FLIM imaging

Bright-field time-lapse images and FLIM data of *in vitro* cultured embryos were collected every 4 hours for a period of approximately 60 hours starting from the 2-cell stage (E1.5) until the blastocyst stage (E4.5). All FLIM images were collected using the Zeiss LSM710 confocal microscope within stage incubator to obtain the normal *in vitro* culture conditions (37 °C, 5% CO_2_) (Movie S1).

### Distance analysis program

The FLIM data collected from individual embryos are placed in either of two categories the H (control group has FLIM signature from the embryos developed to the blastocyst stage) and UH (sample group has FLIM signature from the embryos arrested at compaction stage or even earlier). The distance algorithm can generate a “spectra” from the given (up to 24 parameters) of phasor FLIM distributions corresponding to individual embryos. The 24 parameters are the 2 coordinates for the center of mass g and s, 2 second axial moments a and b after diagonalization, the angle of the distribution from the diagonalization and the total number of pixels in the phasor plot from the 4 slices of the 3D phasor histogram. For each parameter set, we calculate the average of the parameters and the standard deviation. Then we construct a function that we call “distance” in which we calculate the difference of the average of the two sets weighted by the variance of the parameter in each set for the group H and UH respectively.

Using distance analysis, a training set can be generated based on the best weight set that has been chosen to separate the H and UH set embryos according to the distance from the average of each set. Finally, after the training set has been generated, the rest of the embryos were tested, and an embryo viability index (EVI) is calculated for each embryo. Using the EVI index for the spectra of the training set, we can build the histogram and determine if a member is a true positive (below 0) or a false positive (above 0). Statistical methods such as the area under the curve (AUC) are then used to determine the quality of the training set. If the AUC is close to one, the two groups are more separable since there are less false positives. The Distance approach has been used previously to determine the separation of spectra in human cancer tissues^33^. More details of the distance analysis calculation can be found in Dr. Ranjit’s recent publication (LFD)^33^.

### Statistical analysis

Data are presented as mean ± standard deviation. For the FLIM data, the statistical analyses were performed using student t-test for the g value only, p < 0.05 was considered as statistically significant.

The box-whisker plot showing the prediction ability represents the median ± min/max from each indicated group (Training set H and UH group, Tested H and UH group).

## Supplementary information

Supplementary Information and Figures

3D Third Harmonic Generation (THG) images of the representative embryos from different pre-implantation embryos.

Supplementary Movie 1: 3D Third Harmonic Generation (THG) images of the representative embryos from different pre-implantation embryos.

Supplementary Movie 1: 3D Third Harmonic Generation (THG) images of the representative embryos from different pre-implantation embryos.

Supplementary Movie 1: 3D Third Harmonic Generation (THG) images of the representative embryos from different pre-implantation embryos.

Supplementary Movie 1: 3D Third Harmonic Generation (THG) images of the representative embryos from different pre-implantation embryos.

Supplementary Movie 2: 60-hour time-lapse imaging of pre-implantation embryos from E1.5 to E4.0.

## Data Availability

The datasets generated during and/or analyzed during the current study are available from the corresponding authors on reasonable request.
