# Supplementary material for: Label-free assessment of pre-implantation embryo quality by the Fluorescence Lifetime Imaging Microscopy (FLIM)-phasor approach
Source: Sci Rep. 2019 Sep 13;9:13206. doi: 10.1038/s41598-019-48107-2 (PMC6744410; doi:10.1038/s41598-019-48107-2)
Supplement: Supplementary file 1 — Supplementary Information and Figures [file 41598_2019_48107_MOESM1_ESM.docx]

**Label-free assessment of pre-implantation embryo quality by the Fluorescence Lifetime Imaging Microscopy (FLIM)-phasor approach**

Ning Ma^†^, Nabora Reyes de Mochel^†^, Paula Duyen Pham^†^, Tae Yeon Yoo, Ken WY. Cho, and Michelle A. Digman

**Supplementary Materials:**


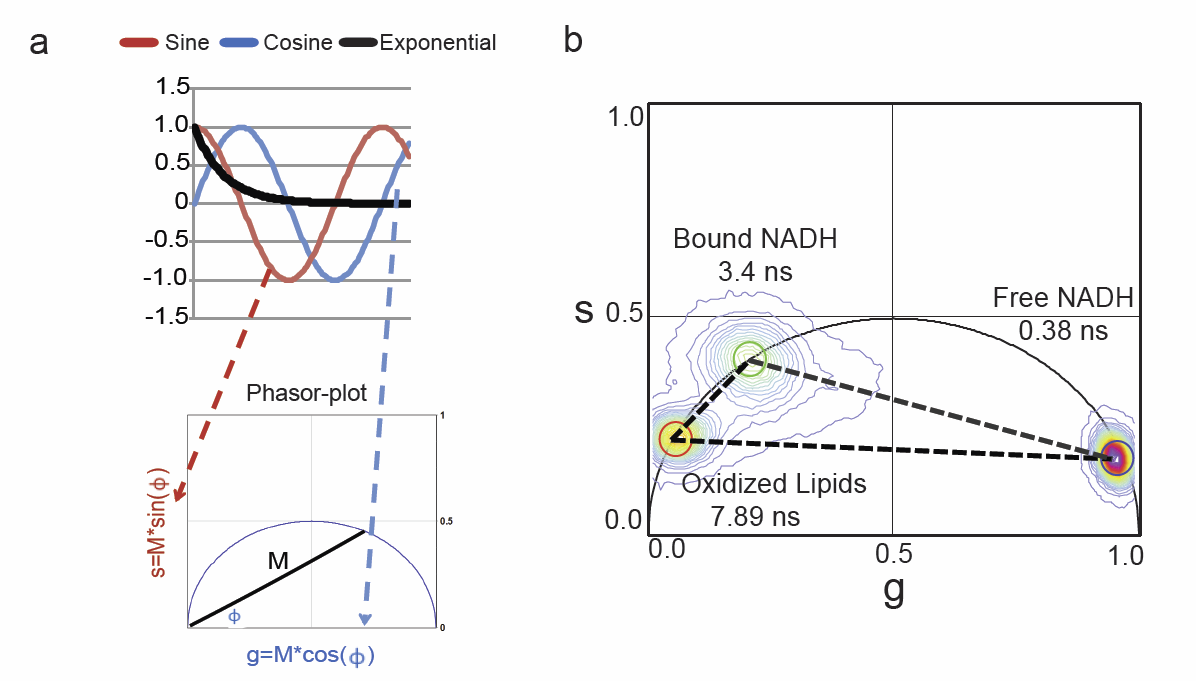


**Supplementary Figure 1: Phasor FLIM analysis.**

**(a)** Phasor FLIM analysis. During FLIM collection, a pulsed 2-photon laser is used to measure the intensity at short time windows (time arrival of the photons) as a function of time. Instead of fitting the decay curve into an exponential equation (black line), the raw data (intensity at each pixel) is transformed into polar coordinates by plotting the sine (red line) and cosine (blue line) using Fourier transformation, for every pixel in the object, the fluorescence lifetime can be obtained as “phasor lifetime” **(b)** Phasor fingerprint of pure intrinsic biomarkers of free NADH in solution, bound NADH in the presence of lactate dehydrogenase, and a long lifetime species derived from lipid droplets. Given that the free form of NADH exhibits a compact structure with a low fluorescence quantum yield (ϕ=0.019) and a short lifetime of 0.4ns and the extended form of NADH bound to lactate dehydrogenase with a much higher quantum yield (ϕ= 0.099) with a longer fluorescence lifetime up to ~3.4ns, the lifetimes of these two states can be easily distinguished(*40*). Based on the law of phasor addition, any sample containing the combination signature of these three species will fall within the triangle joining the three phasors.

**
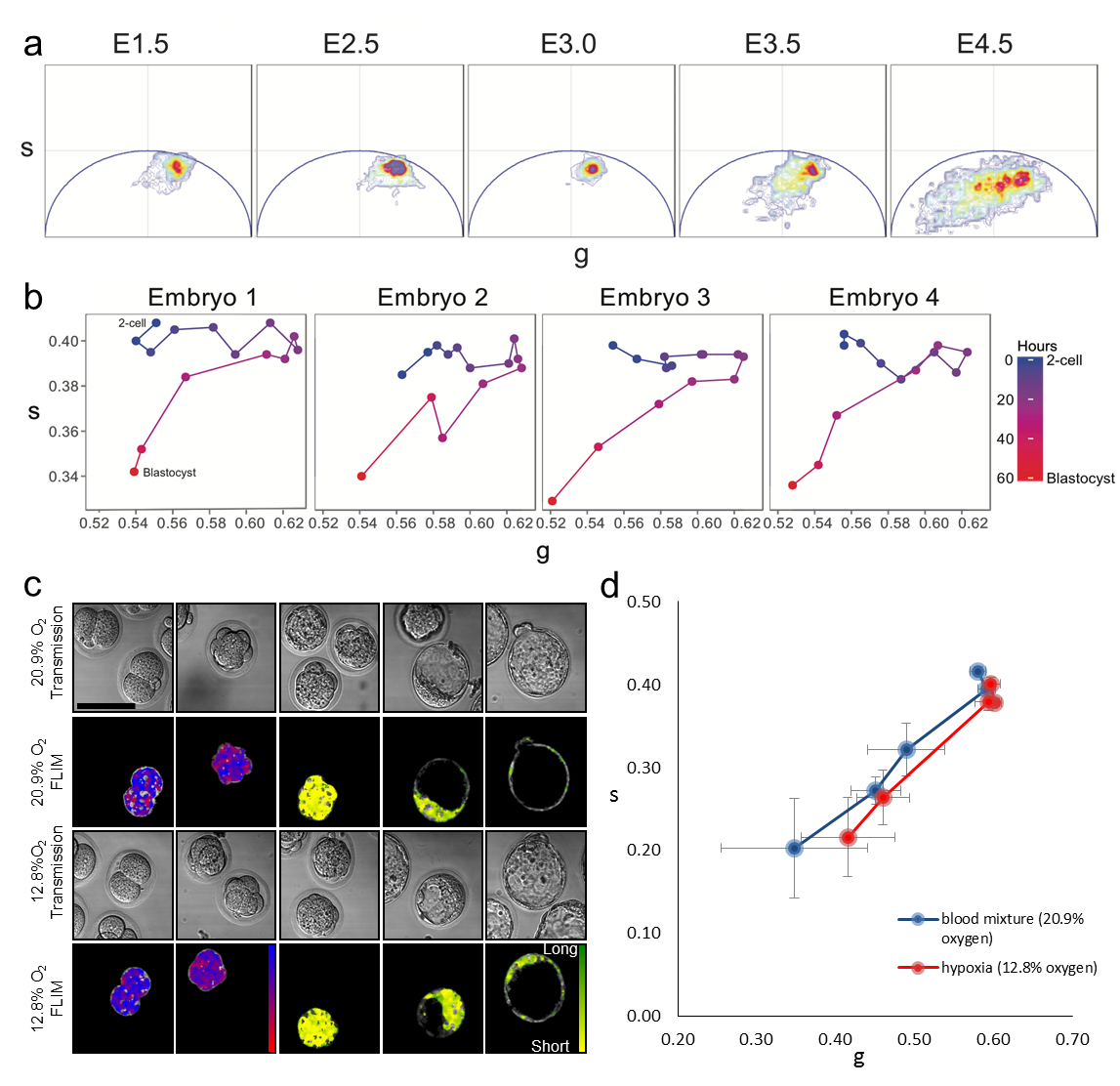
**

**Supplementary Figure 2: Development trajectory composed of two distinct trajectories that correlate with the metabolism of embryonic stages.**

**(a)** Representative phasor plots for the pre-implantation mouse embryo from early cleavage stage to blastocyst stage (E1.5-E4.5). **(b)** Four examples of the D-Trajectory observed throughout pre-implantation stages (from 2-cell to blastocyst stages). **(c)** Transmission and FLIM image plots for pre-implantation mouse embryos from early cleavage stage to blastocyst stage under 20.9% oxygen and 12.8% oxygen conditions. **(d)** D-Trajectory shows the same trend under an hypoxic condition (12.8% Oxygen, red) (n=19, 16, 11, 15, 13 for E1.5, E2.5, E3, E3.5, E4.5 stage respectively) and regular blood mixture culture condition (20.9% oxygen, blue) (n=29, 8, 11, 14, 11 for E1.5, E2.5, E3, E3.5, E4.5 stage, respectively). The straight lines show the phasor-FLIM patterns of developing embryos. The starting scatter plots (representing E1.5 embryos) is in the top right. The last scatter (E4.5) of the lines is in the bottom left. For g values of hypoxia treatment group, compare with regular blood mixture treatment group, p-value=0.296, 0.018, 0.829, 0.488, 0.584 for E1.5, E2.5, E3, E3.5, E4.5 stage, respectively (student t-test, two-tail test for g). The error bars show the standard deviation for each condition. Scale bar set to 100µm.

*
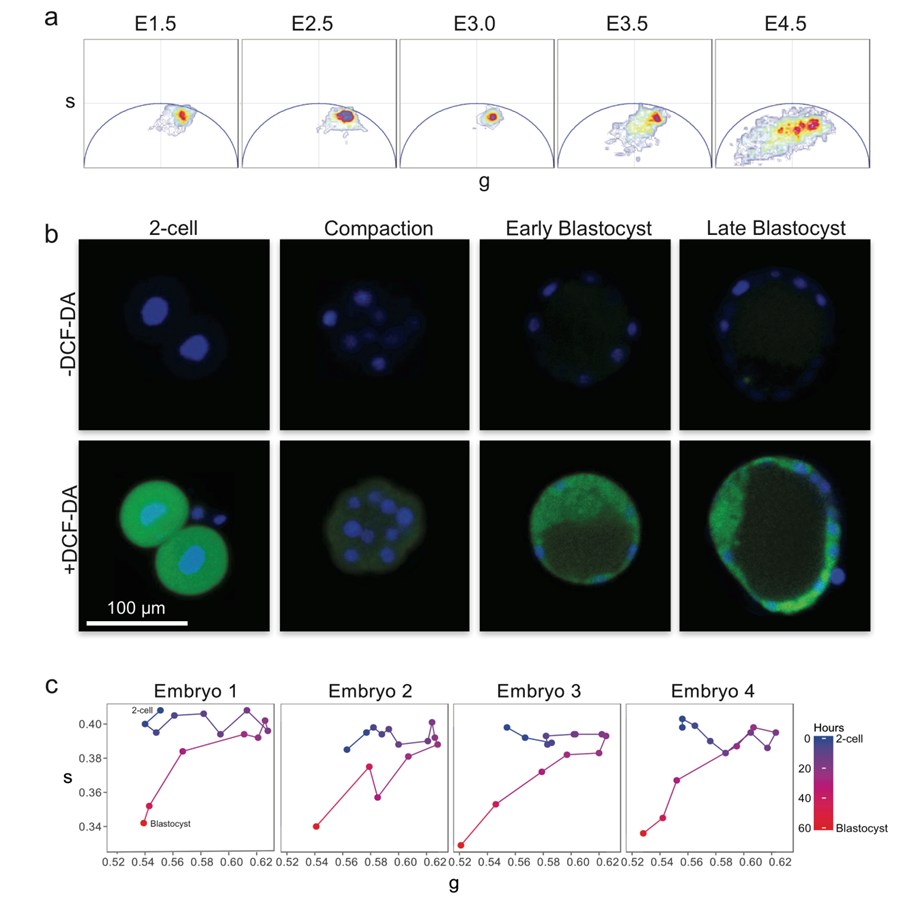
*

**Supplementary Figure 3:** Detection of reactive oxygen species in pre-implantation mouse embryos. 2', 7’-dichlorodihydrofluorescein diacetate (DCF-DA) stain across pre-implantation mouse embryos.


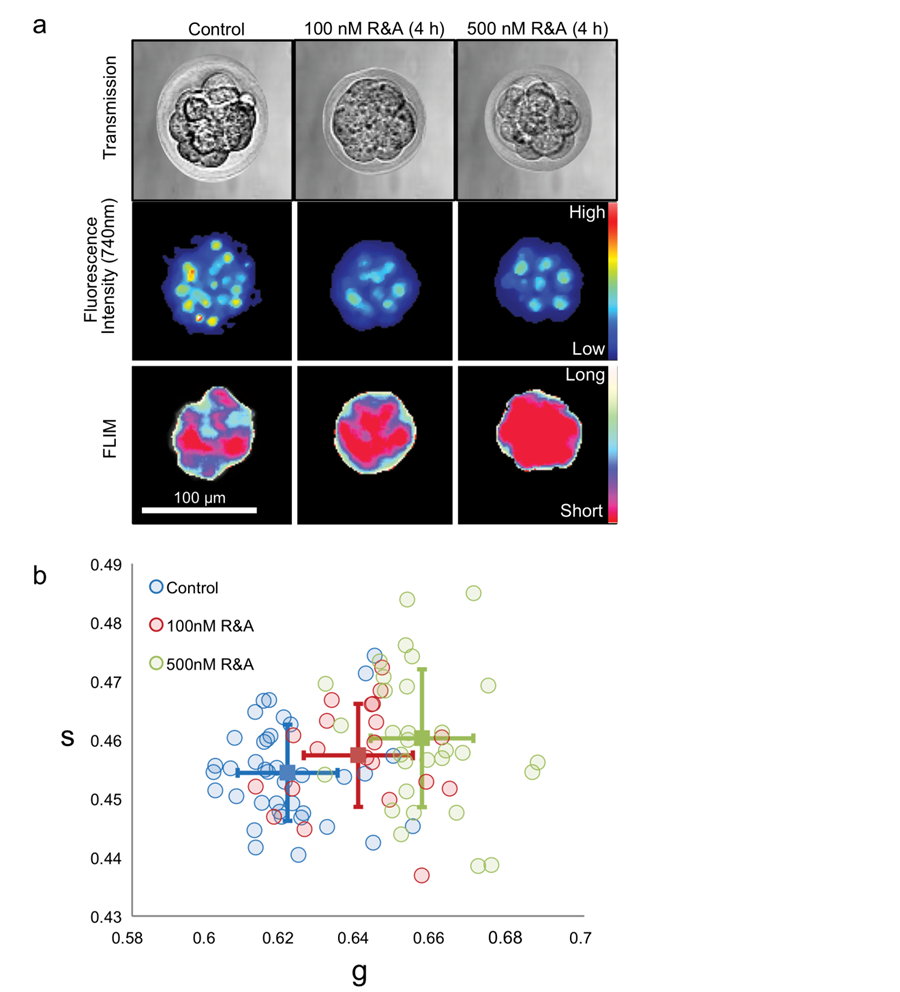


**Supplementary Figure 4: Fluorescence lifetime trajectories reveal dose-dependent metabolic state changes of pre-implantation mouse embryos**. **(a)** Transmission (top), fluorescence (middle) and FLIM (bottom) images for control and 4-hour 100nM and 500nM rotenone and antimycin A (R & A) treated embryos, indicating a shift from long to short lifetimes. **(b)** g and s values of control and 4-hour 100nM and 500nM R&A-treated embryos for individual embryos. Blue circles are controls (n= 38), red circles are 4-hour 100nM R&A-treated embryos (n= 21), and green circles are 4-hour 500nM R&A-treated embryos (n=31). The average of each group can be found in the solid squares (for g value of 100nM treatment group and 500nM group compared with control group, p-value=1.76E-5, and 2.86E-16, respectively). FLIM images indicate a rightward shift from long to short lifetimes. Student t-test and two-tail tests were performed.

*
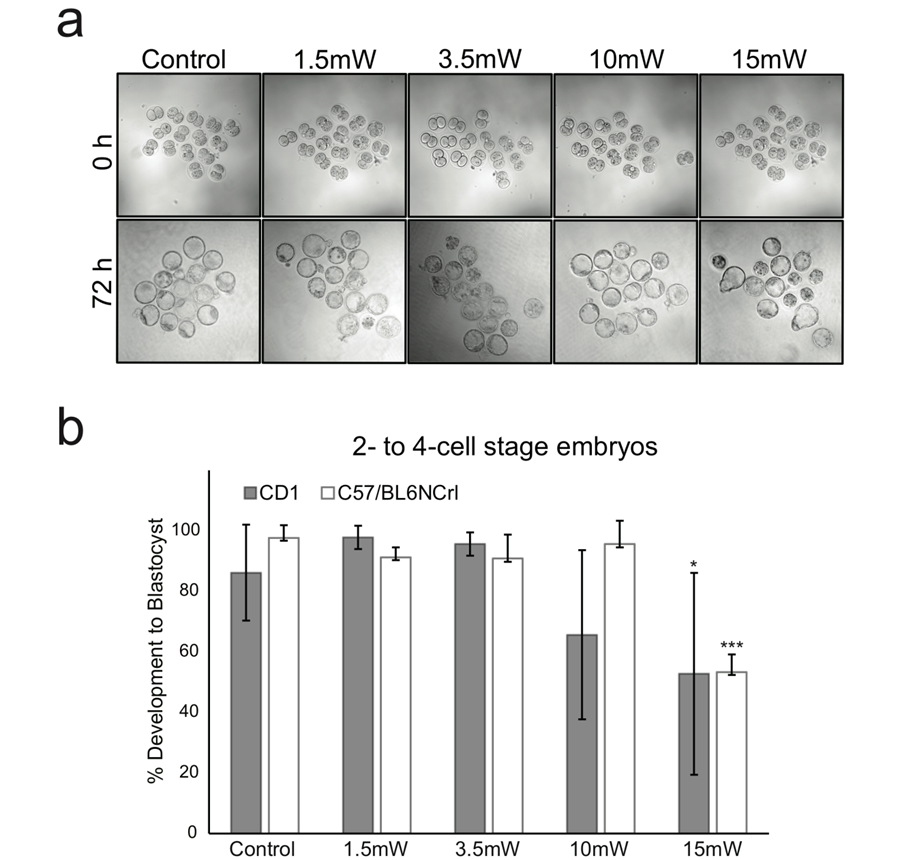
*

**Supplementary Figure 5: Validating the safety of FLIM-imaging of E1.5 pre-implantation mouse embryos. (a)** Transmission images of embryos before and after imaging and culturing for 72 hours till E4.5. Image size is 708.49*708.49 µm. **(b)** Assessment of embryonic development after imaging and 72-hour *in vitro* culture reported as percent development. CD1: E1.5 non-imaged control (n=72); 1.5mW (n=42); 3.5mW (n=42), 10mW (n=31), 15mW (n=45) imaged embryos. C57BL/6NCrl: E1.5 non-imaged control (n=37); 1.5mW (n=37); 3.5mW (n=36), 10mW (n=38), 15mW (n=37) imaged embryos.


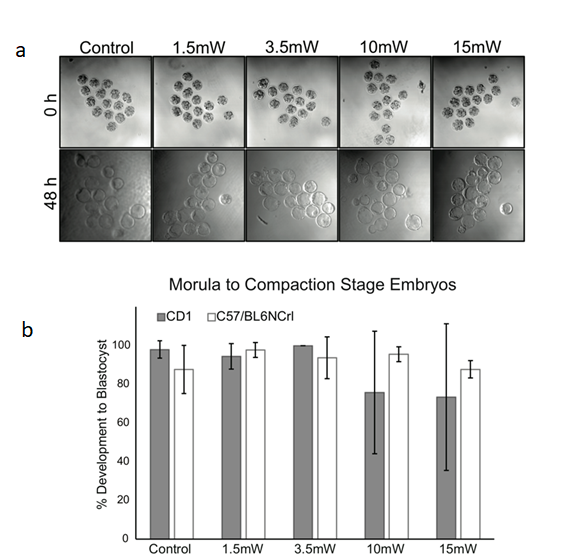


**Supplementary Figure 6: Validating the safety of FLIM-imaging of E2.5 pre-implantation mouse embryos. (a)** Transmission images of embryos before and after imaging and culturing for 48 hours till E4.5. Image size is 708.49*708.49 µm. **(b)** Assessment of embryonic development after imaging and 48-hour *in vitro* culture reported as percent development. For CD1: E2.5 non-image control (n=53); 1.5mW (n=43); 3.5mW (n=43), 10mW (n=28), 15mW (n=28, p<0.05 *) imaged embryos. For C57/BL6NCrl: E2.5 non-image control (n=75); 1.5mW (n=42); 3.5mW (n=47), 10mW (n=44), 15mW (n=41 p<0.001 ***) imaged embryos. Student t-test and one tailed test were performed. Everything is non-significant except the ones annotated.


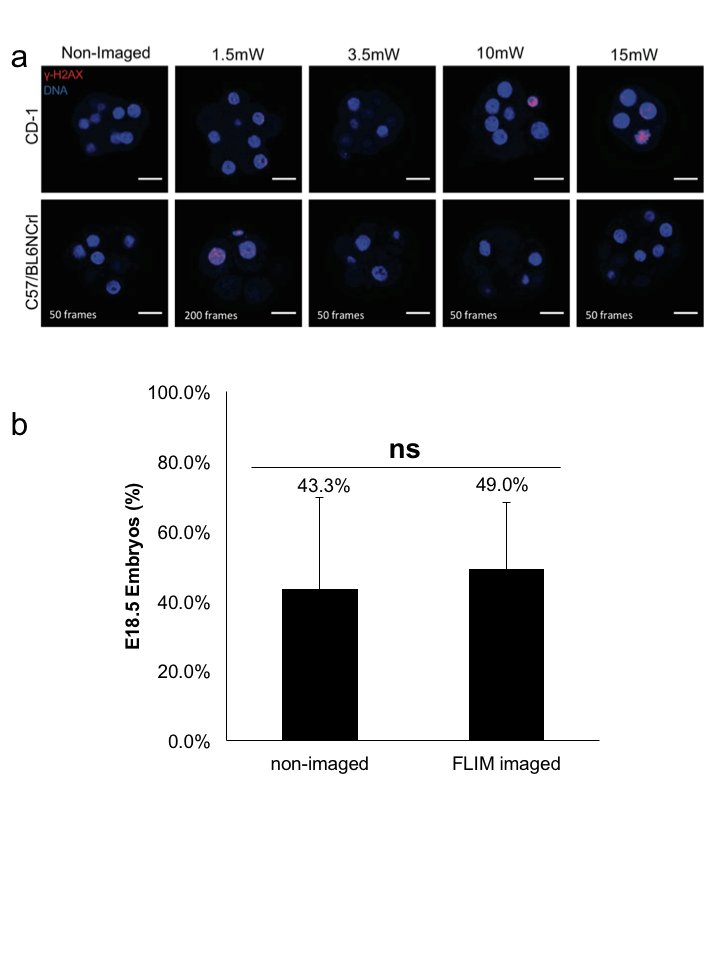


**Supplementary Figure 7: FLIM does not affect live birth rates. (a)** H2AX staining of FLIM imaged pre-implantation embryos. Two strains of embryos were FLIM imaged at E.1.5 and subjected to Hoechst (blue) and H2AX (red) staining at E2.5 for DNA-damage assessment. Control: no FLIM-imaged embryos. For CD1: E2.5 control (n=3); 1.5mW (n=3); 3.5mW (n=3), 10mW (n=3) and 15mW (n=3). For C57/BL6NCrl: E2.5 control (n=9); 1.5mW (n=9); 3.5mW (n=8), 10mW (n=6), and 15mW (n=8). Scale bar set to 20µm. **(b)** Live birth rates of FLIM-imaged embryos. Control (non-imaged, left bar, n=88) and FLIM-imaged CD1 embryos (FLIM imaged, right bar, n=94) were allowed to develop to blastocysts and implanted into 13 pseudo pregnant females (3 independent trials). The pups were collected from C-section on E18.5. There is no statistically significant difference for the live birth rate (p-value, 0.662).


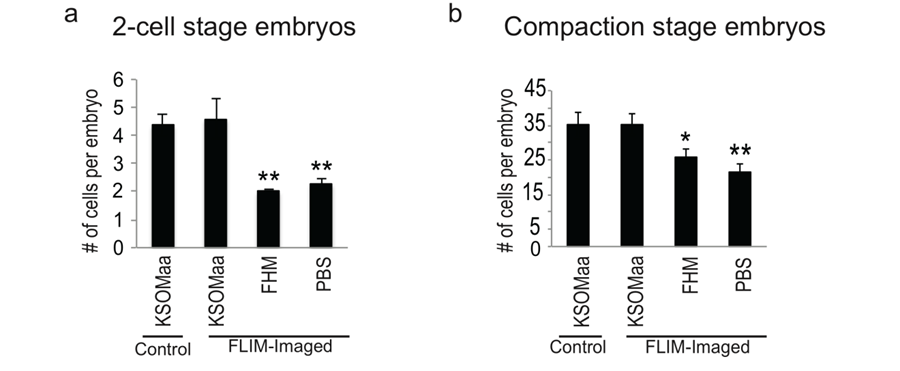


**Supplementary Figure 8: Average number of cells per embryo cultured under high stress conditions. (a)** Bar graph showing the average number of cells present in an embryo after xx hours of culturing in the indicated media starting at the 2-cell stage. KSOMaa non-image control (n=8), KSOMaa (n=10), FHM (n=11), PBS (n=11), compare to the results from KSOMaa group, p-value = 0.004** and 0.001** for FHM and PBS, respectively. **(b)** Bar graph showing the average number of cells per embryo after continuous culturing of embryos in the indicated media starting at the morula stage. KSOMaa non-image control (n=10), KSOMaa (n=18), FHM (n=11), PBS (n=11), p-value = 0.002** and 0.02* for FHM and PBS, respectively.


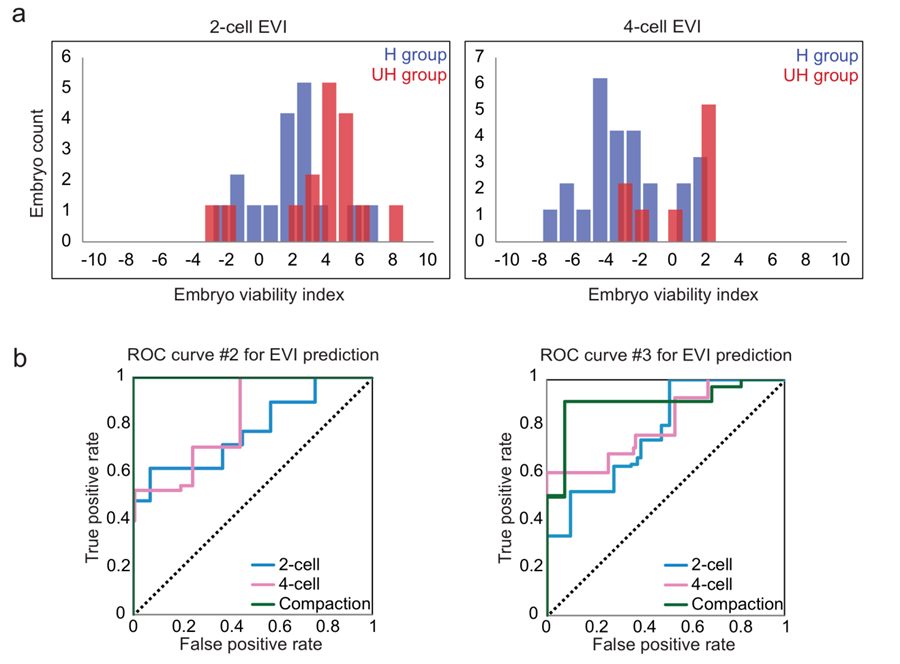


*
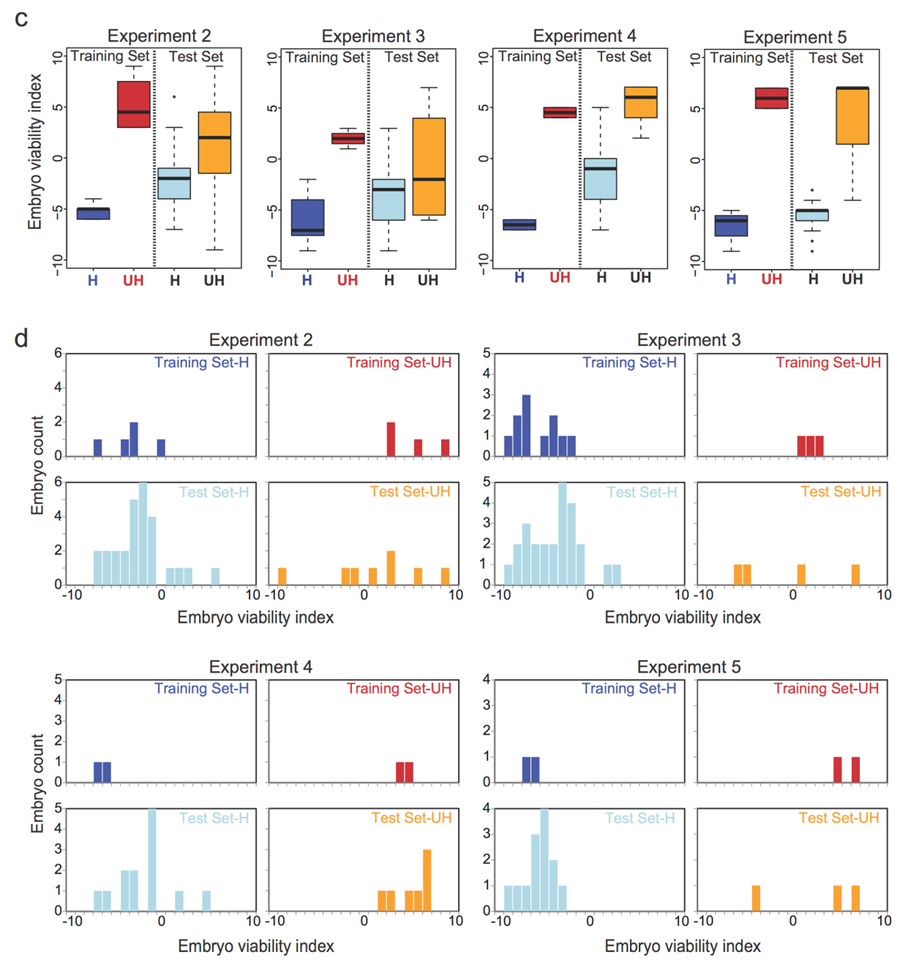
*

**Supplementary Figure 9: Embryo viability index of morula shows the potential to distinguish healthy and unhealthy pre-cleavage stage embryos. (a)** Histogram of embryo viability index of 2-cell and morula stage embryos from one representative experiment (2-cell EVI: H group, (n=18), UH group, (n=17). 4-cell EVI, H group, (n=25), UH group, (n=9)). Each blue and red bar represents the morula stage FLIM-fingerprints of healthy (H) and unhealthy (UH) embryos at 60 hours after imaging respectively. **(b)** Receiver operating characteristic (ROC) curve shows the performance of the binary classification model developed from lifetime distribution patterns of pre-compaction stage embryos (2-, 4-, and early compaction) of two time-lapse FLIM tracking experiments. The area under curve for each stage is 0.777 (2-cell, H n=37; UH, n=8), 0.823 (4-cell, H, n=45; UH, n=8) and 1.000 (early compaction, H, n=30; UH, n=2) for experiment 2, and 0.777 (2-cell, H, n=38, UH, n=10), 0.813 (4-cell, H, n=39, UH, n=7) and 0.945 (early compaction, H, n=39, UH, n=6) for experiment 3.(**c)** Box-whisker plots showing training sets of healthy (H) and unhealthy (UH) groups and predication performance on tested embryos for 4 additional experiments Training set H in navy, Training set UH in red, predicated healthy in light blue, and predicated unhealthy in orange. The n number for the training set healthy group was n= 5, 11, 2 and 3. The n number for the training set unhealthy group was n= 4, 3, 2, and 2. The n number for the test unknown healthy

*
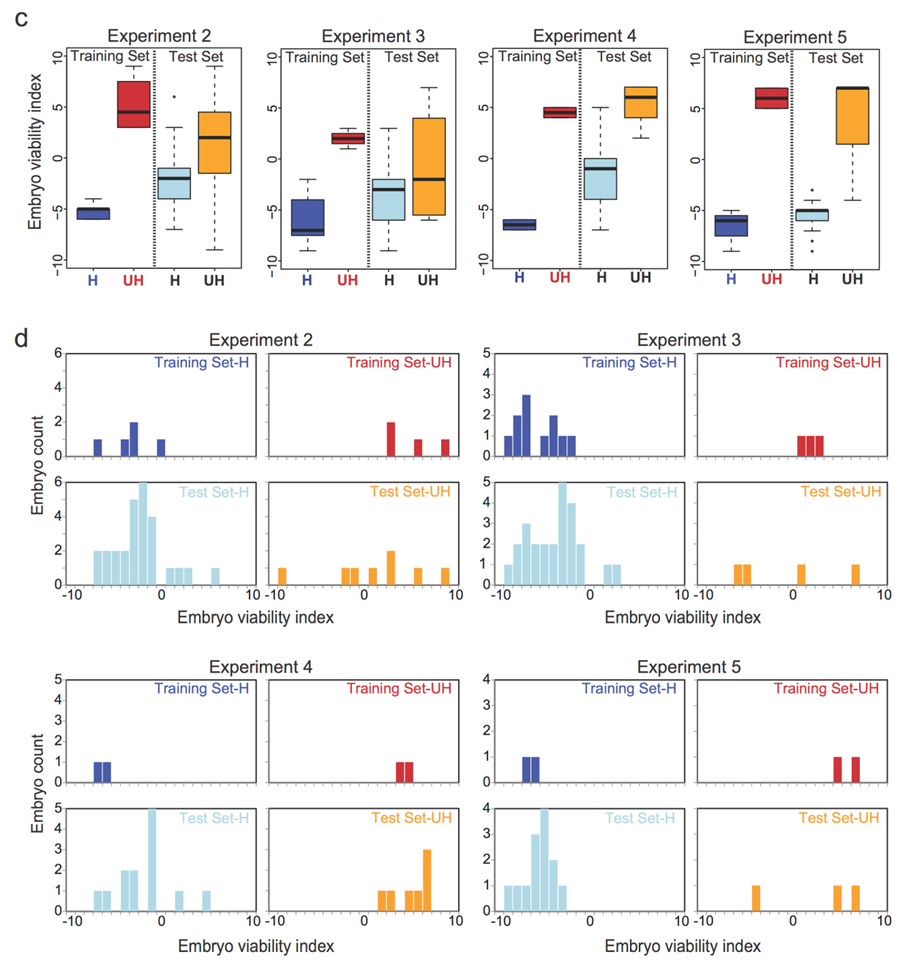
*

**Supplementary Figure 9 (continued):** group was n= 27, 25, 13, and 13. The n number for the test unknown unhealthy group was n= 8, 4, 7, and 3. **(d)** Bar graph of embryo viability index of experiment 2, 3, 4 and 5. Training set H in navy, Training set UHs in red, Healthy in light blue, and Unhealthy in orange.

**Table 1. Implantation Efficacy at E18.5**

| Trial 0 | | | | |
| --- | --- | --- | --- | --- |
|  | CD-1 Embryos Implanted | Bre-gal^+-^ Embryos Implanted | CD-1  E18.5 | Bre-gal^+-^  E18.5 |
| **Mouse 1** | 7 | 7 | 5/7 (71.4%) | 4/7 (57.1%) |
| **Mouse 2** | 7 | 7 | 1/7 (14.3%) | 1/7 (14.3%) |
| **Mouse 3** | 7 | 7 | 4/7 (50%) | 2/7 (25%) |
| **Mouse 4** | 7 | 7 | 1/7 (12.5%) | 4/7 (50%) |
| **Total** | **28** | **28** | **11/28 (39.3%)** | **11/28 (39.3%)** |
| Trial 1 | | | | |
|  | CD-1 Embryos Implanted | Bre-gal^+-^ Embryos Implanted | CD-1  E18.5 | Bre-gal^+-^  E18.5 |
| **Control 1** | 8 | 8 | 4/8 (50%) | 6/8 (75%) |
| **Imaged 1** | 8 | 8 | 6/8 (75%) | 2/8 (25%) |
| **Imaged 2** | 8 | 8 | 2/8 (25%) | 6/8 (75%) |
| **Imaged 3** | 8 | 8 | 1/8 (12.5%) | 2/8 (25%) |
| **Imaged 4** | 8 | 8 | 4/8 (50%) | 4/8 (50%) |
| **Total Control** | **8** | **8** | **4/8 (50%)** | **6/8 (75%)** |
| **Total Imaged** | **32** | **32** | **13/32 (40.6%)** | **14/32 (43.8%)** |

| Trial 2 | | | | |
| --- | --- | --- | --- | --- |
|  | CD-1 Embryos Implanted | Bre-gal^+-^ Embryos Implanted | CD-1  E18.5 | Bre-gal^+-^  E18.5 |
| **Control 1** | 10 | 2 | 1/10 (10%) | 1/2 (50%) |
| **Control 2** | 10 | 2 | 1/10 (10%) | 0/2 (0%) |
| **Control 3** | 10 | 2 | 5/10 (50%) | 1/2 (50%) |
| **Imaged 1** | 10 | 2 | 5/10 (50%) | 2/2 (100%) |
| **Imaged 2** | 10 | 2 | 5/10 (50%) | 0/2 (0%) |
| **Total Control** | **30** | **6** | **7/30 (23.3%)** | **2/6 (33.3%)** |
| **Total Imaged** | **20** | **4** | **10/20 (50%)** | **2/4 (50%)** |

| Trial 3 | | | | |
| --- | --- | --- | --- | --- |
|  | CD-1 Embryos Implanted | Bre-gal^+-^ Embryos Implanted | CD-1  E18.5 | Bre-gal^+-^  E18.5 |
| **Control 1** | 14 | NA | 12/14 (85.7%) | NA |
| **Control 2** | 14 | NA | 3/14 (21.4%) | NA |
| **Control 3** | 14 | NA | 6/14 (42.9%) | NA |
| **Imaged 1** | 14 | NA | 8/14 (57.1%) | NA |
| **Imaged 2** | 14 | NA | 9/14 (64.3%) | NA |
| **Imaged 3** | 14 | NA | 8/14 (57.1%) | NA |
| **Total Control** | **42** | **NA** | **21/42 (50%)** | **NA** |
| **Total Imaged** | **42** | **NA** | **25/42 (59.5%)** | **NA** |

**Supplementary Table 1: Implantation efficacy for the non-image embryos and FLIM-imaged embryos.** Trial 0 is aiming for the baseline collection to test the implantation efficacy for the BRE-gal and CD-1 embryos. Trials 1-3 test the implantation efficacy of embryos after FLIM-imaging. The total percentage of E18.5 embryos retrieved from c-section for control (non-image) group and imaged group were 43% and 49%, respectively (p-value, 0.662).


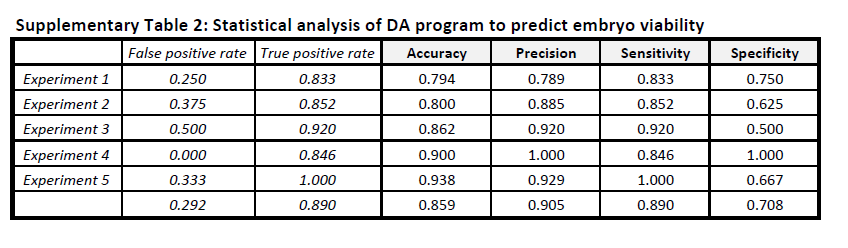


**Supplementary Table 2: Statistical analysis of Distance Analysis (DA) program as a means to predict embryo viability.**

**Supplementary Movie 1: 3D Third Harmonic Generation (THG) images of the representative embryos from different pre-implantation embryos. a) 2-cell, b) 8-cell, c) Compaction, d) Early Blastocyst, e) Blastocyst**

**Supplementary Movie 2: 60-hour time-lapse imaging of pre-implantation embryos from E1.5 to E4.0.** Embryos from H group (blue) are the representative healthy embryos that developed to the blastocyst stage after 60 hours of time-lapse imaging. Embryos from UH group (red) are arrested at late compaction stage.
